# Spinal muscular atrophy caused by a novel *Alu*‐mediated deletion of exons 2a‐5 in *SMN1* undetectable with routine genetic testing

**DOI:** 10.1002/mgg3.1238

**Published:** 2020-04-26

**Authors:** Ivana Jedličková, Anna Přistoupilová, Lenka Nosková, Filip Majer, Viktor Stránecký, Hana Hartmannová, Kateřina Hodaňová, Helena Trešlová, Michaela Hýblová, Peter Solár, Gabriel Minárik, Mária Giertlová, Stanislav Kmoch

**Affiliations:** ^1^ Research Unit for Rare Diseases Department of Pediatrics and Adolescent Medicine First Faculty of Medicine Charles University Prague Czech Republic; ^2^ Department of Clinical Genetics Medirex A.S. Kosice Slovakia; ^3^ Department of Medical Biology Faculty of Medicine P.J. Safarik University Kosice Slovakia

**Keywords:** *Alu* elements, *SMN1*, *SMN2*, spinal muscular atrophy

## Abstract

**Background:**

Spinal muscular atrophy (SMA) is an inherited neuromuscular disease affecting 1 in 8,000 newborns. The majority of patients carry bi‐allelic variants in the survival of motor neuron 1 gene (*SMN1*). *SMN1* is located in a duplicated region on chromosome 5q13 that contains *Alu* elements and is predisposed to genomic rearrangements. Due to the genomic complexity of the *SMN* region and genetic heterogeneity, approximately 50% of SMA patients remain without genetic diagnosis that is a prerequisite for genetic treatments. In this work we describe the diagnostic odyssey of one SMA patient in whom routine diagnostics identified only a maternal heterozygous *SMN1Δ(7–8)* deletion.

**Methods:**

We characterized *SMN* transcripts, assessed SMN protein content in peripheral blood mononuclear cells (PBMC), estimated *SMN* genes dosage, and mapped genomic rearrangement in the *SMN* region.

**Results:**

We identified an *Alu*‐mediated deletion encompassing exons 2a‐5 of *SMN1* on the paternal allele and a complete deletion of *SMN1* on the maternal allele as the cause of SMA in this patient.

**Conclusion:**

*Alu*‐mediated rearrangements in *SMN1* can escape routine diagnostic testing. Parallel analysis of *SMN* gene dosage, *SMN* transcripts, and total SMN protein levels in PBMC can identify genomic rearrangements and should be considered in genetically undefined SMA cases.

## Graphical abstract

Spinal muscular atrophy (SMA) is devastating inherited neuromuscular disease resulting from variants of SMN1 and deficiency of the survival motor neuron protein (SMN). In this work, we describe the diagnostic odyssey for one SMA patient in whom routine diagnostic procedures identified only a heterozygous, maternally inherited deletion of exons 7 and 8 in SMN1. Using an individual approach we found in this case that SMA was caused by a novel Alu‐mediated deletion.

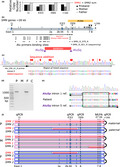

## 1 INTRODUCTION

Spinal muscular atrophy (SMA) is an inherited neuromuscular disease characterized by progressive degeneration of alpha motor neurons in the spinal cord leading to muscle weakness and paralysis. SMA is the most prevalent monogenic cause of death in infancy (Glascock et al., 2018) with an incidence of ~1:8,000 in Caucasians and Asians and carrier frequency of ~1:50 (Verhaart et al., 2017). The severity of SMA can vary from early postnatal onset and muscular weakness with respiratory insufficiency to milder forms presenting during infancy or adolescence (Schorling et al., 2019). The majority of SMA patients carry bi‐allelic variants in the survival of motor neuron 1 gene (*SMN1*, OMIM 600354) that localizes to a duplicated region on chromosome 5q13*.*

The survival of motor neuron 2 gene (*SMN2*, OMIM 601627) is a homologue of *SMN1.* Genetic investigations have revealed zero to six copies of *SMN2* that are located next to *SMN1* on 5q13 (Crawford et al., 2012). *SMN1* and *SMN2* (“*SMN* genes”) encode for the same SMN protein. However, expression of the SMN protein from *SMN2* is substantially lower than from *SMN1* due to a single nucleotide sequence difference at the sixth position of exon 7 in *SMN2* (hg38, chr5:70076526, T). This sequence difference alters splicing and results in the predominant production of an *SMN2* transcript that skips exon 7 (*SMN2Δ7*) and encodes for an unstable and less functional protein. *SMN2* is therefore unable to fully compensate the deficit of *SMN1*. However, SMN expression from *SMN2* may be increased in a gene dose‐dependent manner (Crawford et al., 2012). This *SMN2* dose variance was suggested to have a compensatory effect on SMN expression and ameliorate the severity of SMA (Butchbach, 2016).

The *SMN* region on chromosome 5q13 is enriched for primate‐specific nonautonomous retrotransposons belonging to a class of short interspersed elements (SINE)‐repetitive DNA sequences called *Alu* elements. The *Alu* elements are about 280 base pairs long and are formed by two diverged dimers (Deininger, 2011). They are divided into subfamilies based on single nucleotide differences. The main *Alu* subfamilies are *AluJ*, *AluS*, and *AluY* (Kim, Cho, Han, & Lee, 2016) with the *AluY* being the evolutionarily youngest and *AluS* the most numerous. The youngest *Alu* subfamilies *AluS* and *AluY* increase the likelihood of genomic rearrangements that result in the formation of a new chimeric *Alu‐Alu* element at the breakpoint junction.

*Alu*‐mediated genomic rearrangements are a frequent cause of various human diseases (Song et al., 2018). Accordingly, 95% of genetically defined SMA patients have deletions of exons 7 and/or 8 of *SMN1* (*SMN1Δ7*, *SMN1Δ(7–8)*) that appear to be caused by *Alu*‐mediated rearrangements (Ottesen, Seo, Singh, & Singh, 2017).

It is important to note that the term “deletion of *SMN1* exons 7 and/or 8” is commonly used to describe the results of routinely performed multiplex ligation‐dependent probe amplification (MLPA) assays, which are the current gold standard of SMA diagnostics (Mercuri et al., 2018). Deletions of these two particular exons can be detected by the MLPA assay, which is designed to target exclusively exons 7 and 8 and distinguishes *SMN1* and *SMN2* based on single nucleotide sequence differences. In reality, these deletions can extend beyond exons 7 and 8 and include the entire *SMN*1, then even extending further to include multigene deletions of the 5q13 region*.*

Other *Alu*‐mediated genomic rearrangements in the *SMN* region identified in SMA patients lead to formation of *SMN1*‐*SMN2* hybrid genes (van der Steege et al., 1996). Interestingly, *Alu*‐mediated deletion of exons 4 to 6 with intact exons 7 and 8 has thus far been reported in just a single case (Wirth et al., 1999). Other *SMN1* variants identified in SMA patients include small intragenic deletions and missense variants. A full list of these variants can be found in the Human Gene Mutation Database records (Stenson et al., 2014).

Recently, non‐5q‐*SMN1* variants have been reported in SMA patients, including variants in *VRK1* (Renbaum et al., 2009), *EXOSC3* (Wan et al., 2012), *EXOSC8* (Boczonadi et al., 2014), and *SLC25A46* (Wan et al., 2016). Variants in these genes have been reclassified as a distinct syndrome pontocerebellar hypoplasia (OMIM 607596). Similarly, variants in *AGTPBP1* (Karakaya et al., 2019; Shashi et al., 2018) have also been reported to cause childhood‐onset neurodegeneration with cerebellar atrophy (OMIM 618276), a different type of motor neuron disease.

Due to the complexity of the *SMN* 5q13 genomic region, approximately 50% of all SMA patients remain without a genetic diagnosis after routine genetic testing (Karakaya et al., 2018). The ability to identify the genetic cause of SMA is critically important for patients because only patients with bi‐allelic *SMN1* variants are eligible for genetic therapies (Michelson et al., 2018). These potential treatments include Zolgensma and the antisense oligonucleotide treatment Spinraza. The precise identification of the causal variants in SMA patients is also important for genetic counseling in affected families.

In this work we describe the diagnostic odyssey for one SMA patient and her parents from Slovakia in whom the routine MLPA assay and subsequent direct sequencing of *SMN1* coding regions identified only a heterozygous, maternally inherited deletion of exons 7 and 8 in *SMN1.*

## 2 MATERIALS AND METHODS

### 2.1 Ethical compliance

The study was approved by the appropriate institutional review boards and the investigations were performed according to the Declaration of Helsinki principles. Parents provided informed consent.

### 2.2 Clinical report

The patient was clinically diagnosed at the Children Teaching Hospital Košice, Slovakia. The infant was born by normal spontaneous delivery to a 31‐year‐old mother following a full‐term pregnancy from the first gravidity and with no reported abortions; the postnatal adaptation of the infant was standard. The patient presented with global muscle hypotonia and hyporeflexia suggestive of SMA at the age of 1 month and showed markedly decreased mobility at the age of 3 months. Global respiratory failure required tracheostomy and mechanical ventilation from the age of 7 months. At the time of investigation, the patient was 2 years and 8 months old and ventilator dependent. She suffered from severe global weakness and hypotonia. Muscle atrophy predominantly affected the lower limbs. The patient could respond only with eye contact. There were no sensory deficits. Both parents were neurologically intact.

### 2.3 MLPA, panel sequencing, and cytogenetic analyses

Genomic DNA (gDNA) was isolated from peripheral venous blood in the patient and her parents using standard protocol. The patient’s karyotype was assessed by G‐banding and comparative genomic hybridization (aCGH) was performed using Agilent SurePrint HD 4x44 platform at the Medirex Group, Slovakia. Genes associated with a set of neuromuscular disorders were analyzed using a custom SeqCap EZ kit (Roche) and Illumina sequencing at CMBGT in Brno, Czech Republic. The TruSight One Sequencing Panel (Illumina) was used for analysis of more than 4,800 genes associated with human diseases at Medirex. The presence of the deletion of exons 7 and 8 in *SMN* genes was assessed using the MLPA assay; SALSA MLPA P060 SMA Carrier probemix (MRC‐Holland) at Genexpress. The coding regions of *SMN* genes were analyzed using paired‐end sequencing of PCR amplicons on Illumina MiSeq (Illumina) at Alpha Medical.

### 2.4 *SMN1* and *SMN2* mRNA/cDNA analysis

Total RNA and cDNA were isolated and prepared from peripheral blood mononuclear cells (PBMC) using ProtoScript® II Reverse Transcriptase (NEB). Full‐length *SMN1* and *SMN2* cDNAs were PCR amplified using primers SMN575_F (Sun et al., 2005) and SMN_541C1120_R (Lefebvre et al., 1995) (Table 1) amplifying together both *SMN* genes derived transcripts from the first coding exon to the last untranslated exon 8 (NM_000344.3). PCR products were analyzed using the agarose gel electrophoresis, and Sanger sequenced using the version 3.1 Dye Terminator cycle sequencing kit (ThermoFisher Scientific) with electrophoresis on an ABI 3500XL Avant Genetic Analyzer (ThermoFisher Scientific).

**Table 1:** Primers used for long‐range PCR, qPCR, and *SMN* cDNAs amplification and *Alu* PCR

Primer ID	Primer sequence (5′−3′)	Primers application
SMN1_1U	TTAAGGATCTGCCGCCTTCC	Long‐range PCR, PCR1 (*SMN1*)
SMN_1L	CCAAACCAGCCCACACATTG	
SMN_2U	CTACAGTAGCTGGGGACTGAGC	Long‐range PCR, PCR2
SMN_2L	CATATGGAGGAAACCGGCCTAA	
SMN_3U	CACCATGCCCGGCCTAAAT	Long‐range PCR, PCR3
SMN_3L	CAAGAGCACTGCATCTGGGT	
SMN_4U	AGCCAGGTCTAAAATTCAATGGC	Long‐range PCR, PCR4
SMN_4L	TGGGCCAAAGGGCAAAATAA	
SMN575_F	ATCCGCGGGTTTGCTATG	cDNA *SMN* exons 1–8
SMN_541C1120_R	CTACAACACCCTTCTCACAG	
SMN_i1_105_F	TCCCTATTAGCGCTCTCAGC	qPCR *SMN* region; I3
SMN_i1_182_R	CGGATCGACTTGATGCTGT	
SMN_ei3_7_F	ACAAAATGCTCAAGAGGTAAGGA	qPCR *SMN* region; E3I3
SMN_i3_96_R	TCGGTGGATCAAACTGACAA	
SMN_i5e6_2_F	AAACAATATCTTTTTCTGTCTCCAGAT	qPCR *SMN* region; I5E6
SMN_e6_797_R	GAAATTAACATACTTCCCAAAGCATC	
SMN_28867_F	TGTCCTTGTGGTTGTAAGGAATC	qPCR *SMN* region; +1 kb
SMN_28961_R	CAGCAACTTTTGTCTGTCTTCTG	
Alu_259_wt_R	CCAGGCTGGAGTGCAGTGG	*Alu* PCR
Alu_259_4A_R	CCAGGCTGGAGTGCAATGG	*Alu* PCR
Alu_259_3C_R	CCAGGCTGGAGTGCAGCGG	*Alu* PCR
Alu_259_1A_R	CCAGGCTGGAGTGCAGCGA	*Alu* PCR
SMN_i5_979_R	AACGAGGACGAAAAGACAGC	*Alu* PCR
SMN_i5_821_R	ACAGCTCACATAGCATTTCG	*Alu* PCR sequencing
SMN_i1_10748_F	GGACTTGTCTCACTAATCCCTCAT	Family screening fo*r SMN*(*2a−5)* transcript
SMN_i5e6_1R	GGAGGTGGTGGGGGAATTATC	

### 2.5 Western blot analysis of SMN protein

The quality and amount of the SMN protein were assessed in lysates of PBMC using Western blot analysis. PBMC were isolated using the Histopaque‐1077 reagent (Sigma‐Aldrich). The cell pellet was resuspended in 50 mM Tris pH 6,8, 50 mM DTT, 2% SDS, and Complete Protease Inhibitor Cocktail (Roche), sonicated using the Covaris S2 Ultrasonicator (Covaris), and denaturated at 100°C for 10 min. The protein content in the supernatant was determined using an infrared spectrometer Direct Detect (Millipore) according to the manufacturer's protocol. Protein lysates equivalent to 22 μg of protein were reduced and denatured at 100°C for 10 min in a sample buffer with 1% beta‐mercaptoethanol before SDS‐PAGE electrophoresis. After protein transfer to the polyvinylidene fluoride (PVDF) membrane, the membrane was blocked by 5% skimmed milk and 0.1% Tween 20 in PBS for 1 hr at room temperature (RT). The SMN protein was visualized by incubation with mouse monoclonal SMN antibody (610646, BD Transductions) at 1:5,000 in 5% BSA and 0.1% Tween 20 in PBS over night at 4°C, followed by incubation with goat anti‐mouse HRP (Pierce) at 1:10,000 in 0.1% Tween 20 in PBS for 60 min, and detection was performed by Clarity Western ECL Substrate (Bio‐Rad). The actin protein was visualized by incubation with rabbit Actin antibody (A2103; Sigma‐Aldrich) at 1:1,000 in 0.1% BSA and 0.1% Tween 20 in PBS for 1 hr at RT, followed by incubation with goat anti‐rabbit HRP (Pierce) under conditions and using detection as described above. Relative quantification of the SMN protein was performed using GeneTools software (4.03.03.0, Syngene). SMN protein levels were normalized to actin; the experiment was performed in three technical replicates. The statistical significance was determined using one‐way ANOVA test.

### 2.6 Long‐range PCR

Long‐range PCR was performed using four primer pairs amplifying both *SMN* genes (NG_008691.1, NG_008728.1) in four overlapping PCR products (PCR1‐PCR4, Table 1). The reactions were performed with TaKaRa LA PCR Kit Ver. 2.1 according to the manufacturer`s protocol. PCR products were pair‐end sequenced on Illumina HiSeq 2500 (Illumina). FASTQ files were aligned to the human reference genome hg19 using NovoAlign (V2.08.03) and all alignment locations were reported. Picard Tools (1.129) were used to convert SAM to BAM, remove duplicates, and add read groups. Local realignment around indels, base recalibration, and genotyping was performed with the Genome Analysis Toolkit, GATK (3.5) (McKenna et al., 2010). Variants were annotated by SnpEff (4.3t) (Cingolani et al., 2012) and GEMINI (0.20.2‐dev) (Paila, Chapman, Kirchner, & Quinlan, 2013).

### 2.7 Copy number analysis of *SMN* genes

The total number (sum) of *SMN* genes dosage was determined in the gDNA of the patient and her parents using the quantitative PCR (qPCR) analysis of four retrotransposon‐free *SMN* genomic regions: (a) in intron 1 (I1), (b) in the exon 3–intron 3 junction (E3I3), (c) in the intron 5–exon 6 junction (I5E6), and (d) ~1 kb downstream from exon 8 (+1 kb) (Table 1). The qPCR was performed using the LightCycler® 480 System (Roche Applied Science). All of the qPCR reactions were performed in triplicates in 10 μl reactions with the following final concentrations of the reagents: 80 nM UPL probe, 300 nM primers, 1x Roche Probe Master Mix, and total 15 ng of genomic DNA per reaction. Sample quantitation cycle (cq) values were determined using the Second Derivative Maximum Method and normalized using the RNAse P and albumin genes as a references. Relative quantification using the 2−ΔΔCT method (Livak & Schmittgen, 2001) was performed to determine the sum copy number. Unrelated healthy controls were used as control samples.

### 2.8 Mapping of *SMN1* deletion breakpoint/junction by *Alu* PCR

The *Alu* PCR was performed as described previously by Majer et al. (2020). Briefly, based on the *SMN1* transcript analysis that identified deletion of exons 2a to exon 5 in *SMN1* cDNA, we anticipated that the deletion breakpoints must be located in adjacent introns 1 and 5. We identified in these regions sequences of *Alu* retrotransposons (Figure S1) and designed (a) a set of universal reverse primers targeting the terminal parts of the *Alus* in intron 1 (Alu_259_wt_R, Alu_259_4A_R, Alu_259_3C_R, Alu_259_1A_R) (Table 1, Figure S1), and (b) one *SMN1*‐specific forward primer targeting from intron 5 toward intron 1 (SMN_i5_979_R, Table 1). We performed four separate PCR reactions (Figure S2) with the intron 5 *SMN1*‐specific primer and one of the four *Alu* universal primers. Resulting PCR products of the four reactions were column isolated and Sanger sequenced using a gene‐specific primer located in intron 5 *Alu* preceding region (SMN_i5_821_R) (Table 1). The Sanger sequencing was performed as described above.

The deletion‐spanning PCR method for testing of family members for the Δ2a‐5 deletion was performed using primers annealing to the intron 1 (SMN_i1_10748_F) and exon 6 of *SMN1* (SMN_i5e6_1R, Table 1). Resulting PCR products were column isolated and Sanger sequenced as described above.

## 3 RESULTS

### 3.1 Routine genetic testing in the patient identified only heterozygous, maternally inherited deletion of exons 7 and 8 in *SMN1*

To establish the diagnosis, MLPA analysis was performed and revealed a deletion of *SMN1* exons 7 and 8 and two copies of *SMN2* exons 7 and 8. Heterozygous deletion of *SMN1* exons 7 and 8 was also found in the patient’s mother, who also carried only one copy of *SMN2* exons 7 and 8. The father carried two copies of exons 7 and 8 of both *SMN* genes (Table 2).

**Table 2:** Results of routine MLPA analysis

	*SMN1*		*SMN2*	
	Exon 7	Exon 8	Exon 7	Exon 8
Patient	1	1	2	2
Mother	1	1	1	1
Father	2	2	2	2

Subsequent targeted sequencing of the coding regions of *SMN* genes in the patient and parents did not reveal any further pathogenic variants that would explain the SMA phenotype. To search for other potential disease‐causing variants, karyotype assessment and array‐based comparative genomic hybridization assay (aCGH) were performed but no gross chromosomal abnormalities were detected. The panel sequencing of genes associated with a set of neuromuscular disorders and the TruSight One Sequencing Panel did not reveal any definitive or likely pathogenic variants related to the phenotype. Due to inconclusive results of genetic testing, the patient was referred to the Research Unit for Rare Diseases of the First Faculty of Medicine, Charles University in Prague, and included in the “Undiagnosed Disease Program” that aims to identify genetic diagnosis in cases of rare genetic diseases with negative results of genetic and genomic analyses.

Before looking for other genetic causes, the primary focus was to further analyze the *SMN* genomic region in this patient whose clinical phenotype was highly suggestive of SMA.

### 3.2 Direct sequencing of *SMN* cDNAs revealed an absence of a full‐length *SMN1* transcript in the patient

**Figure 1:**
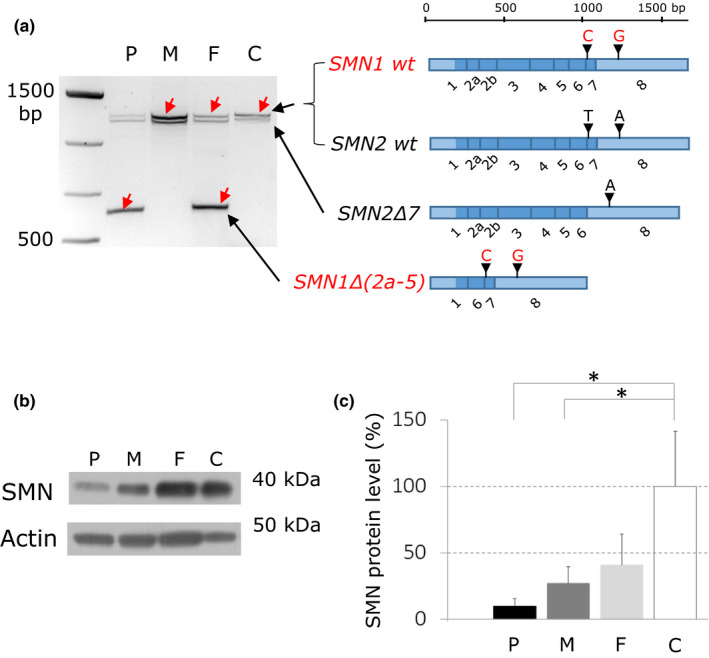
*SMN1* transcript and SMN protein analysis. (a) Agarose gel electrophoresis profiles of RT‐PCR products amplified from total RNA isolated from PMBC of the patient (P), her mother (M), father (F), and control (C) showing presence of the abnormal RT‐PCR product of ~600 base pairs in the patient and her father. Identities of individual RT‐PCR products revealed by Sanger sequencing are shown on the right. (b) Western blot analysis of total PBMC homogenates. Detection with anti‐SMN and anti‐actin antibodies showed presence of immune‐reactive proteins of molecular weights of ~40 and 50 kDa corresponding to predicted molecular weight of the SMN and actin, respectively. (c) The graph shows the relative amounts of SMN normalized to actin and decrease in SMN content in the patient (P), her mother (M), and father (F) compare with control (C). *p < .05

To study the *SMN1* transcript we performed reverse transcription polymerase chain reaction (RT‐PCR) and amplified the full‐length *SMN* cDNAs from PBMC of the patient, both parents and controls. Using the agarose gel electrophoresis, we observed in the patient and her father an abnormal PCR product of ~600 bp that was not present in mother and controls (Figure 1a). Subsequent Sanger sequencing of the gel isolated PCR products revealed the presence of the full‐length *SMN1* and *SMN2* cDNA in the mother, father, and control. In the patient, only the full‐length *SMN2* cDNA was present and the full‐length *SMN1* cDNA was lacking. The abnormal shorter cDNA of ~600 base pairs that was detected in the patient and father was, based on the presence of two *SMN1*‐specific sequence variants in exons 7 and 8, identified as a *SMN1* that was lacking exon 2a to exon 5 (Δ2a‐5) (Figure 1a).

### 3.3 Western blot analysis confirmed decreased amount of SMN protein in PBMC

To analyze the effect of identified mRNA changes on the protein quality and abundance, we immunodetected and quantified SMN protein in PBMC lysates from the patient, her parents, and a control. In all samples we detected only one immune‐reactive protein at ~40 kDa, corresponding to the predicted molecular weight of full‐length SMN (Figure 1b). The protein at the length of ~10 kDa corresponding to a predicted molecular weight of the deleted SMN(Δ2a‐5) was not detected probably due to its altered immunogenicity or reduced protein stability. Compared with controls, the amount of SMN normalized to actin was reduced to 10% in the patient, 27% in the mother, and 41% in the father (Figure 1c).

### 3.4 *SMN1* and *SMN2* gDNA copy number analyses suggested the presence of the intragenic *SMN1* deletion in the patient and father and whole‐gene *SMN1* deletion in the patient and her mother

**Figure 2:**
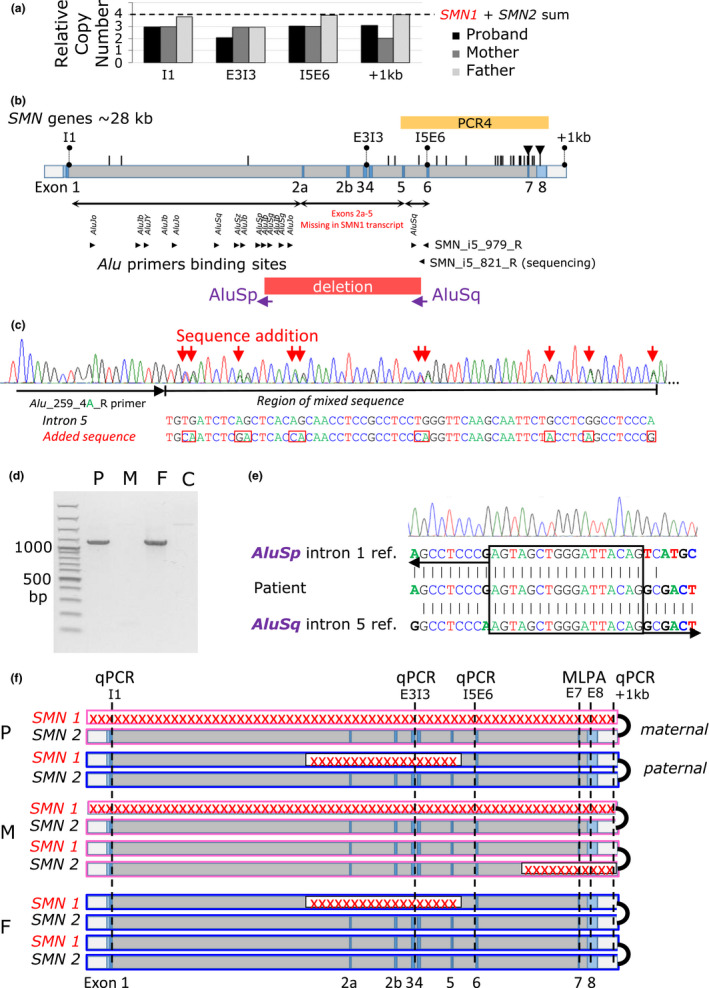
Identification of SMN1 variants. (a) qPCR analysis of four retrotransposon‐free SMN genomic regions in the intron 1 (I1), in the exon 3–intron 3 junction (E3I3), in the intron 5–exon 6 junction (I5E6), and ~1 kb downstream from exon 8 (+1 kb). Compared with four copies of SMN genes that are present in controls, we found that the patient has three copies at the I1, I5E6, and +1 kb loci and two copies at the E3I3 loci. The mother has three copies through the I1 to I5E6 loci and two copies at the +1 kb loci. The father has three copies only at the I5E6 loci. (b) Schematic representation of SMN1/2 exon (E)/intron (I) structure. Positions of sequence differences between SMN1 and SMN2 are represented by black vertical bars. The black triangles denote sequence‐specific variants in exons 7, 8 targeted by MLPA probes in routine testing. Locations of *Alus* in the breakpoint candidate regions in the intron 1 and 5, including the causal *AluSp* in the intron 1 and *AluSq* in the intron 5 indicated by vertical text, and primers binding sites for *Alu* PCR indicated by black arrowheads are shown below the scheme of the *SMN* structure. Position of the PCR4 spanning exons 5–8 that showed absence of *SMN1* sequence‐specific variants indicating disruption of both *SMN1* alleles in the patient is represented by yellow box. Range of the paternal deletion of exons 2a‐5 is represented by red box. (c) DNA sequence trace of the Alu PCR, Alu_259_4A, showing a double sequence caused by presence of *AluSq* wt in intron 5 together with a sequence originating from the intron 1 *AluSp*. Red arrows indicate the addition of *AluSp*‐specific sequence in an *Alu* PCR product. (d) PCR genotyping of the *SMN1Δ(2a‐5)* variant showed presence of the deletion‐spanning amplification product in the patient (P) and father (F), but not in mother (M) and control (C). (e) DNA sequence trace of the breakpoint junction‐specific PCR and detail of the Δ2a‐5 breakpoint junction show the new *Alu*‐*Alu* chimeric element originating from the recombination between the *AluSp* in the intron 1 and *AluSq* in the intron 5. A breakpoint microhomology of the *AluSp* and *AluSq* is marked with a black box. (f) Schematic representation of *SMN1* and *SMN2* in the family members. Pink‐marked boxes represent maternal alleles (M) and blue boxes paternal alleles (F). The red crosses denote identified deletions and the dashed vertical lines denote loci of the qPCR (I1, E3I3, I5E6, and +1 kb) and MLPA (exon 7‐E7, exon 8‐E8) probes used for deletion mapping. The black junctions on the box terminals indicate a cis configuration of *SMN1* and *SMN2* alleles. The model shows (a) a whole deletion of one *SMN1* allele in the patient (P) inherited from her mother and detected by the combination of the qPCR and MLPA; (b) a deletion of the second *SMN1* allele in the patient inherited from her father and detected by the E3I3 qPCR and transcript analysis (Figure 1a); and (c) deletion of one copy of one *SMN2* allele in the mother detected by the MLPA and the + 1kb qPCR

To assess *SMN* gene dosage, we performed qPCR analysis of four retrotransposon‐free *SMN* genomic regions: (a) in intron 1 (I1), (b) in the exon 3–intron 3 junction (E3I3), (c) in the intron 5–exon 6 junction (I5E6), and (d) ~1 kb downstream from exon 8 (+1 kb). Compared with four copies of *SMN* genes that are normally present in each of four tested loci in controls, we found that the patient has three copies at the I1, I5E6, and +1 kb loci and two copies at the E3I3 loci. The mother has three copies through the I1 to I5E6 loci and two copies at the +1 kb loci. The father has three copies only at the I5E6 loci (Figure 2a). Together with the MLPA assay findings (Table 2), this analysis suggested that the patient had deletion of the whole *SMN1* on the maternal allele (NC_000005.9:g.(?_70221078)_(70249850_?)del), where the genomic coordinates denote the central position of the qPCR probes targeting the I1 and +1 kb genomic loci, and a deletion encompassing Δ2a‐5 exons of *SMN1* on the paternal allele.

### 3.5 Sequencing of long‐range gDNA PCR products spanning *SMN* genomic sequence indicated bi‐allelic deletion of the *SMN1* in the patient

To identify the variant causing the deletion of *SMN1* exons 2a‐5 in the patient's and father's cDNA, we PCR amplified the genomic DNA and Illumina sequenced four overlapping long‐range amplicons (PCR1–PCR4) spanning exons 1–8 of *SMN1* and *SMN2*. Using specific single nucleotide sequence differences that distinguish the *SMN* genes*,* we found that in the patient amplicon PCR4, spanning intron 6, exon 7, intron 7, and exon 8 (Figure 2b), had been amplified exclusively from *SMN2* ***.*** These findings indicated that *SMN1* gene must be disrupted on both alleles in the patient.

### 3.6 *SMN1* deletion breakpoint/junction mapping revealed paternal *Alu*‐mediated deletion

To identify the exact nature of the variant on the paternal allele, we considered the *Alu*‐mediated rearrangement of *SMN1* as the most likely mechanism. Using computer analysis we identified in the candidate breakpoint region of intron 1 a set of 21 *Alus* (Figure 2b)*;* in the candidate region of intron 5 we identified only one *AluSq* (Figure 2b). We considered *AluSq* to be a candidate breakpoint start site and anticipated recombination between the *AluSq* and one of the *Alus* located in intron 1 resulting in the formation of a new chimeric *Alu*. With this assumption we performed four PCR reactions using always one of the universal *Alu* primers and the *SMN1* intron 5–specific primer flanking the *AluSq*. Sanger sequencing of obtained PCR products revealed that two of them contained both the wild‐type (*wt*) *AluSq* sequence of intron 5 and the sequence originating from a new chimeric *Alu* resulting from the rearrangement with *AluSp* element originating from the intron 1 (Figure 2c; Figure S2).

Using this sequence we designed and performed a deletion‐spanning PCR allowing for genotyping of the Δ2a‐5 deletion. The deletion‐spanning PCR product was obtained from DNA of the patient and her father, but not from the mother and control (Figure 2d). Sanger sequencing of this PCR product (Figure 2e) defined the new *Alu*‐mediated *SMN1* deletion ranging 8,978 base pairs as NC_000005.9:g.70232118‐70241095del; NM_000344.3:c.82‐2548_723+515del.

### 3.7 Genetic analysis of the *SMN* region correlates with the variance in SMN expression in PBMC

In summary, the genetic analysis (Figure 2f) established that the patient is a compound heterozygote for the ~9 kbp deletion in *SMN1* that she inherited from her father and the deletion of the entire *SMN1* that she inherited from her mother. Maternal and paternal *SMN2* alleles were intact. This genotype correlates with the very low (10% of controls) SMN content in PBMC that must be expressed exclusively from *SMN2* (Figure 2b,c). In addition to the deletion of the entire *SMN1*, the mother also has a deletion of the terminal part of *SMN2*. This correlates with reduced (27% of controls) SMN content in PBMC compared with the father (41% of controls), who has only the ~9 kbp deletion on one of the *SMN1* alleles and both *SMN2* alleles intact.

## 4 DISCUSSION

Spinal muscular atrophy is devastating inherited neuromuscular disease resulting from variants of *SMN1* and deficiency of the survival motor neuron protein (SMN). Several clinical or experimental therapies for SMA augmenting levels of SMN are currently available or in development (Groen, Talbot, & Gillingwater, 2018). The treatment is provided only to individuals with an established genetic diagnosis. Despite great progress in genetic diagnostic methods, approximately half of the patients suspected to have SMA still remain without a genetic diagnosis after routine genetic testing (Karakaya et al., 2018). This unsatisfactory situation is due to the complexity of the *SMN* genomic region and lack of methods allowing routine identification of individual genomic rearrangements.

In this work, we describe the diagnostic odyssey for one SMA patient in whom routine diagnostic procedures identified only a heterozygous, maternally inherited deletion of exons 7 and 8 in *SMN1.* SMN is ubiquitously expressed and detectable in PBMC (Sumner et al., 2006). To identify the other *SMN1* variant in this case, we obtained PBMC from the patient and his parents. In this material we successively assessed *SMN* transcripts, SMN protein content, *SMN* genes dosage, and *SMN* genomic sequence. Using this approach we found in this case that SMA was caused by a novel *Alu*‐mediated deletion encompassing exons 2a to exon 5 (Δ2a‐5) of *SMN1* on the paternal allele and by deletion of whole *SMN1* on the maternal allele.

The (Δ2a‐5) variant of *SMN1* escaped detection by the routine MLPA assay that targets only exons 7 and 8. To the best of our knowledge, this is only the second reported *Alu*‐mediated deletion in *SMN1* that does not encompass the exons 7 and 8 (Wirth et al., 1999).

Our work suggests that other *Alu*‐mediated rearrangements in the *SMN* region that escape detection with routine genetic testing may be more common and should be considered in SMA cases who remain without a genetic diagnosis after standard genetic testing.

We demonstrate that in these cases measurement of SMA protein in PBMC may successfully identify patients with SMA in whom a genetic diagnosis cannot be made. These individuals can then undergo further testing, including *SMN* gene dosage, full characterization of *SMN* transcripts, and precise characterization of the eventual genomic rearrangements. All these parameters are critical for targeted genotyping and eventually for prenatal or preconception analysis in affected families and therapeutic eligibility for affected individuals. We are interested to study other similar cases and can provide the genetic testing and biochemical analyses described here. Please contact ivana.jedlickova@lf1.cuni.cz.

## CONFLICT OF INTEREST

All authors have no conflict of interest to declare.

## AUTHOR CONTRIBUTIONS

IJ, AP, LN, FM, GM, MG, and SK were involved in writing of the manuscript and revised the manuscript. IJ, LN, FM, HH, KHo, HT, KHý, PS, and MG performed the laboratory experiments and interpreted the data. AP, VS, and GM performed bioinformatic studies. MG ascertained the patient and provided the clinical evaluation of the patient. IJ, MG, and SK coordinated the study.

## Supporting information

Fig S1‐S2
