# Supplementary material for: Spinal muscular atrophy caused by a novel Alu‐mediated deletion of exons 2a‐5 in SMN1 undetectable with routine genetic testing
Source: Mol Genet Genomic Med. 2020 Apr 26;8(7):e1238. doi: 10.1002/mgg3.1238 (PMC7336725; doi:10.1002/mgg3.1238)

**Supplementary Figure 1**

**Sequence analysis of *Alu* elements of *SMN1* candidate breakpoint regions in intron 1 and intron 5.** Based on the *SMN1Δ(2a-5)* transcript analysis we supposed an intragenic *Alu* mediated deletion and formation of a new chimeric *Alu* by recombination of two *Alus* of intron 1 and 5. In intron 5 a sole *Alu* element (*AluSq*) was present and located on the minus strand. Since we supposed simple intragenic deletion we listed *Alu* elements located on the minus strand of intron 1. Then we prepared the alignment of *Alu* elements of intron 1 located on the minus strand and the sole *AluSq* of intron 5 (the last in the alignment and in bold). To detect the deletion breakpoint junction (likely within the central part of the chimeric *Alu*) we designed four primers (at the bottom of the alignment) to bind to the terminal part of a newly formed putative chimeric *Alu*. Four PCRs were performed with a primer located in intron 5 *AluSp* preceding region and one of these *Alu* primers targeting *Alu* terminal part.


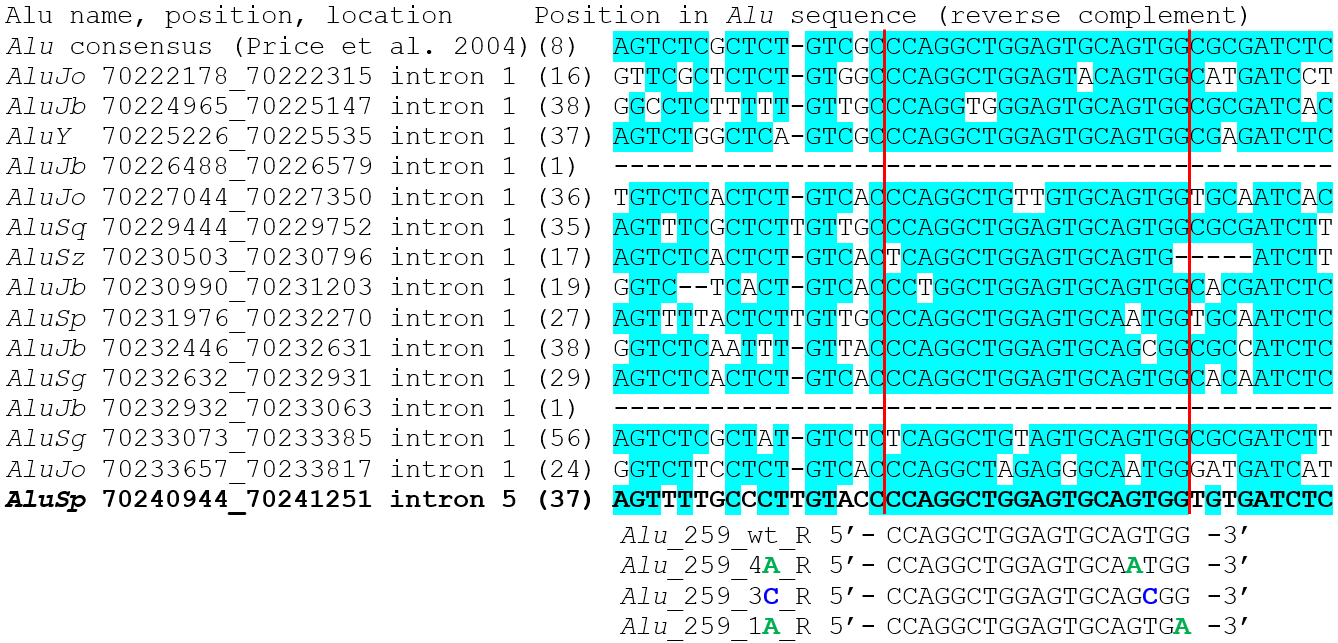


**Supplementary Figure 2**

**Detection of the new chimeric *Alu* using *Alu* PCR. (a)** Agarose gel electrophoresis of the four *Alu* PCR reactions and **(b)** electrophoreograms of the four *Alu* PCRs obtained by sequencing with SMN_i5_821_R primer located in intron 5 *AluSq* preceding region. Two of the four reactions gave deletion spanning PCR products in mixture with *wt* *AluSq* sequence. The red arrows indicate specific additions of a sequence of the new chimeric *Alu* in the two reactions. The deconvoluted sequence of the distal part of the chimeric *Alu* was BLASTed against human genomic database and identified as *AluSp* of intron 1. Bases differing among *Alu* primers used for *Alu* PCRs are indicated by red arrows.

**(a)**


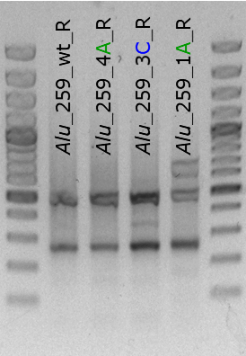


**(b)**


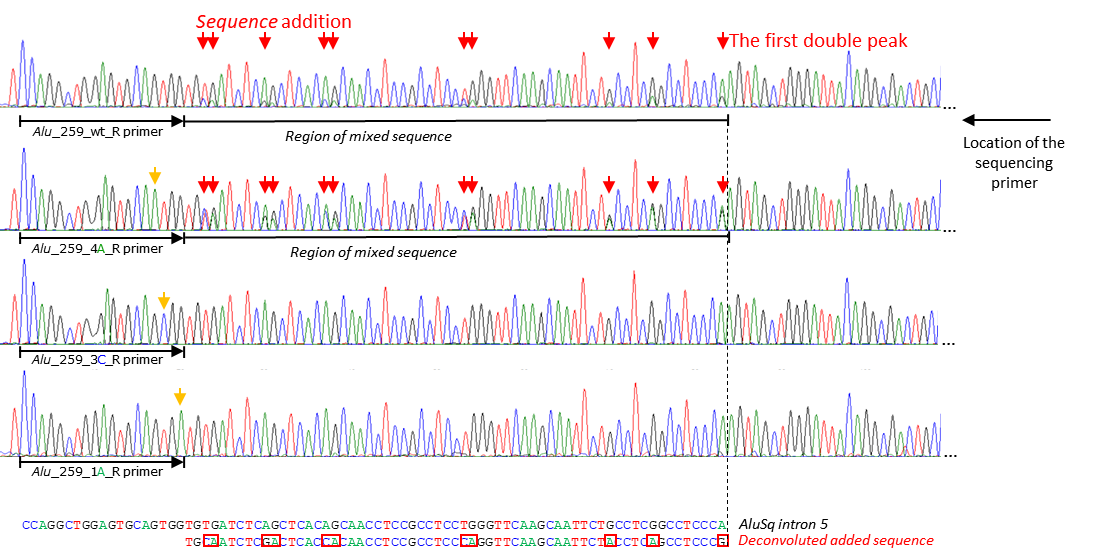

Supplement: Supplementary file 1 — Fig S1‐S2 [file MGG3-8-e1238-s001.docx]
